# Using Multiple Criteria Decision Analysis (MCDA) to make coverage decisions: a methodological framework

**DOI:** 10.1186/2052-3211-8-S1-O7

**Published:** 2015-10-05

**Authors:** Panos Kanavos, Aris Angelis

**Affiliations:** 1 London School of Economics and Political Science, London, WC2A 2AE, UK

## Problem statement

In recent years, multiple criteria decision analysis (MCDA) has emerged as a likely alternative to address shortcomings in health technology assessment (HTA) by offering a more holistic perspective to value assessment and acting as an alternative priority setting tool.

## Objectives

In this abstract we develop a methodological framework and argue that MCDA needs to subscribe to robust methodological processes related to the selection of objectives, criteria and attributes in order to be meaningful in the context of health care decision-making and fulfil its role in value-based assessment (VBA) and as a resource allocation tool.

## Policies targeted

A methodological framework to inform the value assessment of new medical technologies which can help determine coverage decisions and, possibly, pricing mechanisms.

## Stakeholders

Primary and secondary input from a multiplicity of institutional, academic, and other stakeholders (clinicians, methodologists, health economists) under the auspices of the Advance-HTA project consortium.

## Region covered

A generic framework that can be applied across all WHO regions and levels.

## Methods

Study design: Methodological framework development

Time period: April 2014 – April 2015

Setting: Generic framework of value assessment that can be applied to inpatient and outpatient settings in any country

Interventions: Methodological framework development

## Results

We propose a methodological process comprising five distinct phases (problem structuring, model building, model assessment, model appraisal and development of action plans), outline the stages involved in each phase and discuss their relevance in the HTA process. Additionally, criteria and attributes need to satisfy a set of desired properties, otherwise the process of preference elicitation can produce spurious results. The resulting MCDA output, which can take the form of a single universal value index, can be informed by stakeholder participation and therefore can be robust and reflective of stakeholder preferences.

## Conclusions

Assuming the methodological process we propose is adhered to, the application of MCDA presents two very distinct advantages to decision-makers in the context of HTA and VBA: first, it acts as an instrument for eliciting preferences for a wider set of criteria leading to a more complete assessment of value; and, second, the process of preference elicitation is informed by direct stakeholder participation. Both features contribute to greater rationality and increased transparency in decision-making.

